# Restoring Microbial Balance: Clinical Applications, Challenges, and Future Directions of Fecal Microbiota Transplantation in Pediatric Disorders

**DOI:** 10.3390/microorganisms14061241

**Published:** 2026-05-31

**Authors:** Giulia Zambelli, Marco Masetti, Sonia Rasmi, Irene Addati, Lorenzo Bonacorsi, Sonia Diona, Susanna Esposito

**Affiliations:** Pediatric Clinic, Department of Medicine and Surgery, University of Parma, 43126 Parma, Italy; giulia.zambelli@unipr.it (G.Z.); marco.masetti@unipr.it (M.M.); sonia.rasmi@unipr.it (S.R.); irene.addati@unipr.it (I.A.); lorenzo.bonacorsi@unipr.it (L.B.); sonia.diona@unipr.it (S.D.)

**Keywords:** fecal microbiota transplantation, pediatric dysbiosis, recurrent *Clostridioides difficile* infection, inflammatory bowel disease, immunocompromised children, microbiome restoration

## Abstract

Fecal microbiota transplantation (FMT) has emerged as a microbiota-directed therapeutic strategy with established efficacy in recurrent *Clostridioides difficile* infection (rCDI) and expanding investigational applications in pediatric medicine. Given the central role of the gut microbiota in immune maturation, metabolic homeostasis, and colonization resistance—particularly during early life—restoring microbial diversity represents a biologically plausible intervention for disorders characterized by dysbiosis. This narrative review critically examines current evidence regarding the indications, efficacy, safety, and practical considerations of FMT in pediatric populations. A structured literature search was conducted across PubMed/MEDLINE, Scopus, Web of Science, and the Cochrane Library from inception through December 2025. Eligible studies included randomized controlled trials, observational studies, systematic reviews, meta-analyses, and guideline statements addressing pediatric FMT. RCDI remains the primary and best-supported indication, with reported success rates exceeding 80% after a single FMT and approaching 90% with repeat procedures. Evidence for other indications—including inflammatory bowel disease (IBD), malignancy-associated CDI, transplant recipients, multidrug-resistant organism (MDRO) decolonization, neurodevelopmental disorders, allergic colitis, and functional gastrointestinal disorders—remains limited and heterogeneous. While short-term remission rates in pediatric ulcerative colitis appear promising, data derive largely from small, non-standardized studies, and long-term efficacy and safety remain insufficiently defined. FMT usage in immunocompromised children, particularly oncology and transplant populations, is controversial due to limited pediatric-specific evidence and theoretical risks. Substantial variability in donor screening, preparation methods, dosing, and administration routes further limits standardization. Currently, FMT should be considered established therapy for pediatric rCDI, whereas other applications require well-designed, multicenter trials with long-term follow-up to clarify safety and clinical benefit.

## 1. Introduction

Fecal microbiota transplantation (FMT) has emerged as a highly effective therapeutic strategy for several gastrointestinal disorders and is increasingly being explored for a broad spectrum of extra-intestinal conditions. Its clinical success—particularly in recurrent *Clostridioides difficile* infection (CDI)—has reinforced the central role of the gut microbiota in maintaining host homeostasis and has positioned targeted microbial modulation as a promising frontier in disease management [1,2].

The human microbiota comprises a complex and dynamic community of microorganisms inhabiting multiple body sites, with the gastrointestinal tract representing the largest and most metabolically active reservoir [3]. This microbial ecosystem plays a fundamental role in preserving health, especially during early life, when microbial communities are not yet fully established. Beginning at birth, the microbiota undergoes a continuous process of ecological succession, progressively developing into a diverse and resilient ecosystem. Achieving this balanced state is essential for numerous physiological functions, including vitamin biosynthesis, fermentation of complex dietary carbohydrates, regulation of bile acid and host hormone metabolism, maintenance of colonization resistance against pathogens, and orchestration of immune system development and maturation [4,5].

Beyond metabolic contributions, microbial communities exert profound effects on host immune function. A stable and diverse microbiota enables immune tolerance toward commensal organisms while preserving effective defense against opportunistic and pathogenic species [6]. Within the gastrointestinal tract—an anatomical site with substantial potential for microbial translocation—the microbiota serves as a critical barrier against invasive infection and systemic dissemination, including sepsis. Specific microbial taxa, particularly those belonging to the phyla *Bacteroidetes* and *Firmicutes*, produce short-chain fatty acids (SCFAs) that modulate immune homeostasis by influencing regulatory T-cell gene expression and enhancing macrophage antimicrobial activity, thereby strengthening host defense mechanisms [7,8].

The pediatric microbiome differs from that of adults, with early-life colonization shaped by factors such as mode of delivery and maternal microbial transmission [9,10,11]. This dynamic process evolves over the first years of life toward a more stable, adult-like microbial community. Although maturation trajectories vary among individuals, the infant gut microbiota is generally considered highly dynamic from birth through approximately 2–3 years of age, after which microbial diversity, stability, and functional capacity progressively converge toward an adult-like configuration; therefore, infants and toddlers ≤3 years should be considered separately from older children and adults when interpreting microbiota-dependent interventions [10,11].

Dysbiosis, defined as an imbalance in the composition, diversity, or function of the gut microbial ecosystem, is increasingly recognized as a key contributor to disease pathogenesis [12]. Systemic antibiotic exposure, a frequent occurrence in pediatric practice, can markedly reduce microbial diversity, impair colonization resistance, and deplete commensal-derived antimicrobial peptides, thereby facilitating pathogen overgrowth and immune dysregulation [13,14,15,16].

FMT is defined as the transfer of fecal material from a healthy donor to a recipient with the aim of restoring a balanced and functional gut microbiome [17,18,19,20,21]. Unlike conventional probiotic formulations, FMT delivers a complex consortium of microorganisms, metabolites, and microbial-derived proteins, thereby recapitulating the ecological and functional diversity of a healthy intestinal ecosystem [22]. Given this complexity, rigorous donor screening is essential to minimize the risk of transmissible infections. Although universally standardized protocols are lacking, major American professional societies—including the Infectious Diseases Society of America (IDSA), the American Society for Gastrointestinal Endoscopy, the North American Society for Pediatric Gastroenterology, Hepatology and Nutrition, and the American College of Gastroenterology—advocate comprehensive, multimodal donor evaluation strategies [17,19].

Donor selection may follow a patient-directed approach or rely on centralized stool banks employing universal donors with standardized recruitment and screening procedures [23]. Screening typically includes an initial pre-assessment questionnaire addressing medical history, lifestyle factors, and potential exposure risks not detectable through laboratory testing, followed by same-day eligibility confirmation prior to donation [20]. Notably, the use of age-matched donors has been associated with a lower incidence of adverse events [21].

FMT material can be prepared from fresh stool or from frozen preparations stored at −80 °C following aliquoting with cryoprotectants [18]. Administration routes include both upper and lower gastrointestinal approaches—oral capsules, nasoenteric infusion, enemas, and colonoscopic delivery. Colonoscopy remains the most extensively studied route, whereas upper gastrointestinal administration has been associated with a higher frequency of adverse events [18,23,24]. Although generally well tolerated, FMT is not devoid of risk; abdominal discomfort is the most commonly reported adverse event [25].

While recurrent CDI remains the most established indication for FMT, its potential applications have expanded to include inflammatory bowel disease (IBD), constipation and inflammatory bowel syndrome, allergic colitis, neurodevelopmental and neuropsychiatric disorders such as autism spectrum disorder, and decolonization of multidrug-resistant organisms (MDRO) [24,26,27,28]. These emerging indications reflect growing recognition of dysbiosis as a pivotal contributor to pediatric disease pathophysiology. However, despite increasing clinical interest, the evidence supporting FMT in children remains heterogeneous, and important questions persist regarding safety, long-term outcomes, optimal protocols, and patient selection—particularly in vulnerable populations such as immunocompromised children.

Therefore, the aim of this narrative review is to critically examine the current evidence regarding the indications, efficacy, safety profile, and practical considerations of fecal microbiota transplantation in pediatric disorders, with particular emphasis on recurrent CDI, immunocompromised populations, inflammatory bowel disease, and other emerging clinical applications, while identifying existing knowledge gaps and future research priorities.

## 2. Methods

Although this work is structured as a narrative review, a systematic literature search was conducted to ensure comprehensive identification of relevant evidence regarding FMT in pediatric populations.

A structured search of the literature was performed in the following electronic databases: PubMed/MEDLINE, Scopus, Web of Science, and the Cochrane Library. The search covered the period from database inception through December 2025 to ensure inclusion of both historical and the most recent data.

The search strategy combined Medical Subject Headings (MeSH) terms and free-text keywords related to FMT and pediatric populations. Boolean operators (AND, OR) were used to optimize sensitivity and specificity. Core search terms included combinations of: “fecal microbiota transplantation,” “*Clostridioides difficile* infection,” “immunocompromised,” “inflammatory bowel disease,” “irritable bowel syndrome,” “indications,” “epidemiology,” “neonates,” “infants,” “children,” and “pediatric.” No language restrictions were applied.

To enhance completeness, the reference lists of all included studies and relevant reviews were manually screened to identify additional eligible publications not captured by the initial database search.

Eligible studies included: randomized controlled trials; prospective and retrospective cohort studies; case–control studies; large case series; systematic reviews and meta-analyses; official reports, consensus statements, and clinical guidelines issued by recognized health authorities. Studies were included if they addressed at least one of the following aspects in pediatric populations: indications and eligibility criteria for FMT; epidemiology and clinical characteristics of diseases treated with FMT; safety and efficacy outcomes; real-world or time-dependent outcome data; special pediatric subpopulations (e.g., immunocompromised children, oncology patients, transplant recipients) not fully represented in major guideline recommendations. Original pediatric studies, including randomized controlled trials, prospective and retrospective cohort studies, case–control studies, and large case series, were prioritized for extraction of efficacy and safety outcomes. Systematic reviews, meta-analyses, consensus statements, and clinical guidelines were not pooled with original studies to avoid duplication of patient populations or overestimation of treatment effects; instead, these documents were considered separately to contextualize recommendations, define clinical endpoints, and compare the available evidence with current practice guidance.

For the purposes of this review, the pediatric population was defined as patients from birth to <18 years of age, including neonates, infants, children, and adolescents. When available, age-specific data were extracted separately, with particular attention to infants and toddlers ≤3 years of age, given the developmental immaturity and distinct composition of the gut microbiota in this age group compared with older children and adults.

Exclusion criteria were: narrative reviews, commentaries, editorials, or opinion articles without original data or systematic methodology; case reports or case series including fewer than five patients, unless they provided novel epidemiological insights or relevant antimicrobial resistance data; studies lacking sufficient methodological details to allow assessment of study design, patient selection, or outcomes.

Expected clinical endpoints were predefined according to the indication for FMT. For recurrent CDI, primary endpoints included clinical resolution of diarrhea and CDI-related symptoms without recurrence during follow-up, microbiological testing being interpreted only in the appropriate clinical context, in line with pediatric CDI guidance and FMT position statements. For IBD, endpoints included clinical response or remission, reduction in disease activity scores, corticosteroid-free remission when reported, endoscopic or biomarker improvement, relapse rate, and adverse events, consistent with pediatric IBD trial outcomes and current recommendations that FMT remains investigational outside refractory cases or research settings. Microbiota restoration, including increased microbial diversity or donor-strain engraftment, was considered an exploratory mechanistic endpoint rather than a stand-alone measure of clinical efficacy.

Two independent reviewers (GZ and MM) screened titles and abstracts to assess eligibility. Full-text articles were subsequently retrieved and evaluated according to the predefined inclusion and exclusion criteria. Discrepancies between reviewers were resolved through discussion and consensus. When necessary, a third reviewer (SR) was consulted to adjudicate disagreements. The study selection process was documented by recording the number of records identified through database searches and manual reference screening, the number of duplicates removed, the number of titles/abstracts screened, the number of full-text articles assessed for eligibility, and the final number of selected publications. A total of 113 publications were finally included in the review.

## 3. Clinical Applications of Fecal Microbiota Transplantation in Pediatric Disorders

### 3.1. Clostridioides Difficile Infection

Among microbiota-associated infections, CDI represents one of the most extensively characterized conditions and exemplifies the clinical consequences of intestinal dysbiosis in pediatric populations.

*Clostridoides difficile* is an anaerobic, spore-forming, Gram-positive bacillus whose pathogenicity is primarily mediated by toxin production, particularly toxins A (TcdA) and B (TcdB). These toxins inactivate Rho-family GTPases, leading to cytoskeletal disruption, breakdown of tight junctions, epithelial apoptosis, and mucosal inflammation [29,30]. Hypervirulent strains, such as BI/NAP1/027 and ribotype 078, produce increased quantities of toxins and are associated with more severe clinical manifestations and higher complication rates [30,31].

Over the past two decades, the incidence of CDI in children has risen in both hospital and community settings. Population-based studies from the United States report at least a doubling of pediatric CDI-related hospitalizations between 1991–2009 and 2001–2006 [28,32,33,34]. Estimated incidence rates reach several dozen cases per 100,000 children annually, with substantially higher rates among those with underlying comorbidities, including oncology patients, transplant recipients, and children with inflammatory bowel disease (IBD). Chronic illness, immunocompromised status, and IBD in particular significantly increase both primary CDI risk and recurrence rates [34,35,36] (Table 1).

Reported CDI prevalence and hospitalization trends should be interpreted in light of the diagnostic methods used, which have changed over time. Diagnostic approaches include toxigenic culture, which remains highly sensitive but is labor-intensive and less commonly used in routine clinical practice; enzyme immunoassays or immunochromatographic assays for toxins A and B, which are widely used because of their rapid turnaround but have variable sensitivity; glutamate dehydrogenase screening; and nucleic acid amplification tests (NAATs), which detect toxigenic *C. difficile* genes but may not distinguish active infection from asymptomatic colonization [37]. Therefore, part of the apparent increase in pediatric CDI-related hospitalizations may reflect improved recognition, broader testing availability, and greater use of sensitive molecular diagnostic methods rather than a true rise in disease burden alone. Similarly, reported colonization rates in infants are strongly influenced by diagnostic strategy, sampling timing, and whether studies detect viable organisms, toxins, or toxigenic genes; this is particularly important because asymptomatic carriage is common in early life and toxin detection or molecular positivity does not necessarily indicate clinically significant CDI in infants [37].

CDI most commonly develops following disruption of the intestinal microbiota, typically as a consequence of antibiotic exposure. Antibiotics diminish microbial diversity and colonization resistance, enabling expansion of toxigenic *C. difficile* strains. The resulting toxins damage the colonic epithelium and trigger inflammatory responses that manifest clinically as diarrhea and colitis.

Pediatric CDI exhibits distinctive epidemiological characteristics, particularly the high prevalence of asymptomatic colonization during infancy and the progressive emergence of symptomatic disease in older children [37]. Colonization rates vary considerably by age. Studies assessing colonization in the first week of life report rates near 0% during the initial two days, followed by a gradual increase toward the end of the first week and subsequent weeks of life [38,39,40]. Colonization may reach up to 70% in infants, likely reflecting immaturity of the gut microbiota and limited epithelial toxin receptor expression [41]. Importantly, the infant intestine appears relatively resistant to the pathogenic effects of toxins A and B, and symptomatic infection is uncommon in this age group.

Between 12 and 24 months of age, the transient carrier state typically resolves as the gut microbiota matures toward an adult-like configuration. At this stage, increased susceptibility to toxin-mediated epithelial injury may lead to clinical manifestations resembling adult pseudomembranous colitis (Table 2). Symptomatic CDI is increasingly recognized in older children, particularly in healthcare-associated contexts, although community-acquired cases are also rising [42].

Antibiotic exposure in pediatric patients transiently suppresses colonization by *C. difficile* and other commensals; however, it simultaneously exacerbates microbial imbalance by further reducing protective taxa and enabling opportunistic pathogens to proliferate. Patients with CDI exhibit marked alterations in gut microbial composition, characterized by reduced species richness and diminished overall diversity compared with healthy controls. Recolonization by commensal organisms typically occurs within 2–4 weeks after antibiotic discontinuation [43].

Both molecular and culture-based analyses have demonstrated that active CDI is associated with significant depletion of *Firmicutes*, *Bacteroidetes*, and *Actinobacteria*, accompanied by expansion of *Proteobacteria* [44]. These compositional shifts correlate with reduced butyrate production—an SCFA essential for epithelial barrier integrity and immune regulation—and increased abundance of lactic acid-producing bacteria, thereby fostering a pro-inflammatory intestinal milieu (Table 3).

The high prevalence of asymptomatic colonization in infancy is likely multifactorial. The neonatal gut microbiota, often enriched in *Bifidobacterium*, promotes anti-inflammatory immune responses. Additionally, increased abundance of *Ruminococcus* species may attenuate toxin-mediated pathogenicity. Host factors—including immature epithelial toxin receptors and the presence of passively acquired or endogenously produced toxin-neutralizing antibodies—further contribute to protection against symptomatic disease [44]. Nevertheless, the precise microbial configurations distinguishing asymptomatic carriage from clinically overt infection remain incompletely understood.

Given the central role of dysbiosis in CDI pathogenesis, microbiota-targeted interventions have gained increasing attention. Approaches aimed at restoring microbial diversity and functional balance include probiotics, prebiotics, postbiotics, synbiotics, administration of non-toxigenic *C. difficile* strains, and FMT, all of which have demonstrated varying degrees of efficacy in pediatric populations [44,45]. The 2023 World Gastroenterology Organisation (WGO) guidelines recommend *Saccharomyces boulardii* (250–500 mg) for reducing the risk of *C. difficile*–associated diarrhea in children [45]. Prebiotics and synbiotics may further support microbial recovery by promoting the growth and metabolic activity of beneficial commensal bacteria, while postbiotics exert immunomodulatory and barrier-protective effects through bioactive microbial metabolites [13,44]. In addition, colonization with non-toxigenic *C. difficile* strains represents a promising strategy to competitively inhibit toxigenic strains and reduce recurrence risk [44]. Furthermore, two microbiota-based therapeutic products have recently received U.S. Food and Drug Administration approval for prevention of recurrent CDI in adults [46,47]. Although pediatric data are currently lacking, these therapies aim to restore microbial diversity and enhance secondary bile acid production, thereby inhibiting *C. difficile* spore germination and reinforcing colonization resistance [22,43].

Collectively, these findings underscore the pivotal role of gut microbiota composition and function in pediatric CDI pathogenesis and provide the biological rationale for microbiota-directed therapeutic strategies.

### 3.2. Recurrent Clostridioides Difficile Infection

Recurrent CDI (rCDI) is defined as the reappearance of symptoms accompanied by a positive *C. difficile* test within 60 days of completing therapy for a prior CDI episode. Recurrence may reflect either relapse caused by the original infecting strain or reinfection with a genetically distinct strain [30,48]. In pediatric populations, recurrence complicates 12% to 25% of CDI cases [48].

The pathophysiology of rCDI is multifactorial. Persistent intestinal dysbiosis plays a central role, as incomplete restoration of microbial diversity impairs colonization resistance and allows ongoing susceptibility to toxigenic *C. difficile*. In parallel, inadequate humoral immune responses to toxins A and B may limit effective neutralization of bacterial virulence factors, further predisposing to repeated disease episodes [30,49]. Together, these mechanisms create a cycle in which microbial imbalance and immune dysfunction perpetuate recurrence.

Several risk factors have been identified for rCDI in children. These include prior or repeated antibiotic exposure, recent surgery, underlying malignancy, solid organ transplantation, the presence of indwelling devices such as tracheostomy or gastrostomy tubes, use of acid-suppressive therapy, and concomitant administration of non-CDI antibiotics during CDI treatment [50]. Chronic comorbid conditions appear to substantially amplify vulnerability. A large study conducted in 2008 involving approximately 4000 pediatric patients hospitalized with a primary diagnosis of CDI found that nearly two-thirds had at least one complex chronic condition, underscoring the contribution of medical fragility to CDI risk and recurrence in children [51].

RCDI represents the principal indication for FMT in pediatric practice [50]. In the joint position statement issued by the North American and European Societies for Paediatric Gastroenterology, Hepatology, and Nutrition (NASPGHAN and ESPGHAN), FMT is incorporated into the recommended therapeutic algorithm for pediatric recurrent CDI [50]. According to these guidelines, FMT may be considered in children with symptom recurrence within 8 weeks following treatment of at least two severe CDI episodes requiring hospitalization, or after three episodes of mild-to-moderate CDI following failure of a vancomycin regimen administered as a prolonged 6–8-week taper, with or without alternative antibiotic therapies [50,52]. Additionally, the position paper suggests considering FMT in cases of moderate CDI unresponsive to standard therapy after at least one week of appropriate treatment, including vancomycin, as well as in severe CDI that remains refractory after 48 h of standard management [50,52]. These recommendations reflect both the high recurrence burden and the limitations of repeated antibiotic therapy, which may further exacerbate dysbiosis.

Emerging real-world evidence supports the efficacy of FMT in pediatric rCDI. Multicenter data from the United States report an overall success rate of 81% following a single FMT administration, increasing to nearly 90% among patients who required a second procedure [53,54]. However, interpretation of FMT efficacy in pediatric rCDI requires caution because published protocols are not fully uniform: FMT has been administered by colonoscopy, nasoenteric tube, enema, or oral capsules, usually after completion of an anti-*C. difficile* antibiotic course with a short washout period, and with variable stool doses, donor selection procedures, preparation methods, and follow-up definitions; therefore, although reported clinical resolution rates are broadly comparable across cohorts, differences in route, timing, dosage, and outcome assessment limit direct comparison of efficacy between studies [17,18,23,50,52,53].

Collectively, current evidence positions FMT as a highly effective therapeutic option for pediatric rCDI, particularly in patients with multiple recurrences or refractory disease. Nevertheless, continued efforts are needed to optimize patient selection, procedural protocols, and long-term safety monitoring in this vulnerable population.

### 3.3. Children with Malignancies and Transplant Recipients

Available evidence indicates that approximately one quarter of pediatric CDI cases occur in children with underlying malignancies [55]. Multiple cancer-related factors contribute to this increased susceptibility, including the malignancy itself, exposure to cytotoxic chemotherapy, frequent use of broad-spectrum antibiotics, and adjunctive supportive therapies [55]. Together, these elements profoundly disrupt immune function and intestinal microbial homeostasis, creating a permissive environment for opportunistic infections, including CDI.

Several biological mechanisms underlie the heightened infection risk observed in pediatric oncology patients. Hematologic malignancies can directly impair lymphocyte function, predisposing children to opportunistic pathogens. Moreover, anticancer treatments—particularly chemotherapy and hematopoietic stem cell transplantation (HSCT)—damage mucosal barriers of the gastrointestinal tract, oral cavity, and skin, facilitating microbial translocation into normally sterile compartments. Common pathogens implicated in these infections include *Enterococcus* spp., *Staphylococcus aureus*, and *Escherichia coli* [56]. In parallel, anticancer therapies induce profound immunosuppression by compromising the function of immune effector cells, including natural killer (NK) cells and T lymphocytes [57].

Importantly, these treatments also induce significant alterations in gut microbiota composition, promoting dysbiosis and expansion of pathobionts [58]. This microbial imbalance, compounded by barrier disruption and immune dysfunction, contributes to diverse infectious manifestations in oncology patients, including bloodstream infections (BSIs), mucositis-associated infections, and gastrointestinal disease [58].

Within this already vulnerable population, CDI represents an additional and serious clinical challenge. Despite the biologic rationale supporting microbiota restoration strategies, current clinical guidelines do not recommend routine use of FMT in pediatric oncology patients, primarily due to the scarcity of robust data—particularly in neutropenic children receiving active anticancer therapy [51].

Nevertheless, FMT has emerged as a potential therapeutic option in selected cases of refractory CDI. Published case reports suggest that FMT is generally well tolerated in pediatric oncology patients; however, clinical responses have been heterogeneous [59]. In one reported series, two of four patients achieved symptom resolution after a single FMT, whereas one patient required up to six procedures because of repeated recurrences [59]. Although limited in scope, these observations indicate potential efficacy while underscoring variability in treatment response.

Recent advances in microbiome research have significantly deepened the understanding of gut dysbiosis in pediatric patients with hematological malignancies, highlighting its broader clinical implications beyond infection risk. Emerging evidence indicates that disruption of intestinal microbial diversity is associated with increased chemotherapy-related toxicity, delayed neutrophil recovery, and heightened susceptibility to bloodstream infections and mucosal barrier injury [60,61]. Moreover, the gut microbiota has been shown to influence the efficacy and toxicity profile of novel immunotherapies, including chimeric antigen receptor T-cell (CAR-T) therapy, as well as outcomes following hematopoietic stem cell transplantation (HSCT), where microbial diversity correlates with reduced incidence of graft-versus-host disease (GvHD) and improved survival [60,61]. These findings underscore the central role of the microbiome as a modulator of host–treatment interactions in pediatric oncology. In this context, FMT represents a biologically plausible strategy not only for the management of rCDI but also as a potential intervention to restore microbial homeostasis, mitigate treatment-related complications, and improve overall clinical outcomes. However, despite this strong mechanistic rationale, clinical evidence supporting FMT in pediatric oncology remains limited and largely restricted to small observational studies, reinforcing the need for well-designed prospective trials to define its safety, optimal timing, and therapeutic impact in this highly vulnerable population.

A particularly relevant and rapidly evolving area of interest is the role of the gut microbiota in GvHD following HSCT in pediatric leukemia patients [56,60]. Increasing evidence demonstrates that reduced intestinal microbial diversity during the peri-transplant period is strongly associated with a higher risk of GvHD, increased treatment-related mortality, and poorer overall survival. Dysbiosis—often exacerbated by conditioning regimens, broad-spectrum antibiotics, and mucosal injury—leads to depletion of commensal taxa responsible for the production of SCFAs, particularly butyrate, which play a critical role in maintaining epithelial barrier integrity and regulating immune tolerance. Loss of these protective microbial functions contributes to intestinal inflammation, enhanced antigen presentation, and activation of alloreactive donor T cells, thereby promoting GvHD pathogenesis [60]. In this context, FMT has emerged as a promising strategy to restore microbial diversity and re-establish metabolic and immunological homeostasis following HSCT. Preliminary studies—primarily in adult populations but increasingly explored in pediatric settings—suggest that FMT may facilitate microbiota reconstitution, reduce GvHD severity, and improve clinical outcomes when used either as a therapeutic or preventive intervention [58,60]. However, evidence in children remains limited, and important questions persist regarding optimal timing, donor selection, and safety in the setting of profound immunosuppression. Consequently, while FMT represents a compelling microbiota-based approach in pediatric HSCT recipients, its use should currently be considered investigational and restricted to specialized centers or clinical trial settings.

A similarly elevated CDI burden has been documented in pediatric solid organ transplant (SOT) recipients. Hospitalized children undergoing SOT exhibit higher CDI incidence rates than the general pediatric population, paralleling trends observed in adults. Evidence from two single-center studies and one multicenter investigation indicates that most CDI episodes in this group occur within the first 6–9 months following transplantation [62,63]. Although the majority of patients achieve clinical resolution without major sequelae, approximately 7.6% require escalation of care, including intensive care unit admission [63].

Additional risk factors for CDI in pediatric SOT recipients include younger age and more severe underlying disease accompanied by multiple comorbidities [64]. Given their cumulative immunologic and clinical vulnerabilities, identification of effective and safe therapeutic approaches remains a critical priority in this population [62]. Although high-quality pediatric evidence remains limited, FMT is increasingly being explored as a potential option for recurrent CDI in transplant recipients [62].

One of the most frequently cited studies in immunocompromised populations is the 2014 multicenter retrospective analysis by Kelly et al., which evaluated FMT for recurrent, refractory, or severe CDI in 80 immunocompromised patients, including five pediatric cases [65]. The cohort encompassed individuals with SOTs, malignancies, HIV/AIDS, and other causes of immunosuppression. An overall cure rate of 78% was reported following a single FMT, with no recurrences observed at 12-week follow-up [65]. Importantly, no deaths or donor-derived infectious complications attributable to FMT were documented [65]. However, the limited pediatric representation and lack of detailed characterization of immunosuppressive status restrict the generalizability of these findings.

Reflecting ongoing uncertainty, the 2018 *Clostridioides difficile* Infection Clinical Practice Guidelines (CPG) strongly advised against routine FMT use in pediatric immunocompromised patients, citing limited efficacy data, absence of evidence in neutropenic children, and procedural considerations such as colonoscopy and bowel preparation [66]. The 2024 CPG update maintained this cautious stance. Among seven newly included studies, only one involved pediatric patients—and none included neutropenic children. Furthermore, six studies reported inconclusive results when fresh or frozen FMT administered via direct intestinal routes was compared with vancomycin at follow-up [66].

The systematic exclusion of pediatric oncology patients from randomized controlled trials reflects concerns regarding increased susceptibility to bacterial translocation and potential donor-derived infections in severely immunocompromised hosts [59]. Although FMT has demonstrated efficacy in adult patients undergoing chemotherapy, pediatric oncology patients represent a distinct clinical population, characterized by differences in treatment intensity, antimicrobial exposure, microbiota disruption patterns, and developmental immune responses [59]. Consequently, current recommendations consider FMT only as a potential therapeutic option after resolution of profound immunosuppression [66] (Table 4).

Notably, by restoring the predominance of *Bacteroidetes* and *Firmicutes* within the intestinal microbiota, FMT may confer theoretical benefits in immunocompromised children by enhancing colonization resistance and potentially reducing intestinal carriage of multidrug-resistant organisms [59].

### 3.4. Inflammatory Bowel Disease

IBD, comprising Crohn’s disease (CD) and ulcerative colitis (UC), encompasses chronic, relapsing immune-mediated disorders arising from a complex interplay among genetic susceptibility, environmental triggers, gut microbiota alterations, and dysregulated mucosal immune responses. Although primarily affecting the gastrointestinal tract, IBD is increasingly recognized as a systemic inflammatory condition associated with extra-intestinal manifestations and significant long-term morbidity [67]. Approximately 25% of IBD diagnoses occur before the age of 20, and the rising incidence in pediatric populations highlights the growing clinical burden during childhood and adolescence [68].

Despite substantial therapeutic advances, sustained disease control remains difficult to achieve in many children. Pediatric patients often require prolonged exposure to corticosteroids, immunomodulators, or biologic agents, and management is further complicated by growth, developmental, psychosocial, and safety considerations that restrict therapeutic flexibility [69].

Given the central contribution of intestinal dysbiosis to IBD pathogenesis, FMT has emerged as a biologically plausible intervention aimed at restoring microbial diversity and functional equilibrium. However, while FMT has demonstrated encouraging signals of efficacy in adult IBD populations, the quality and quantity of pediatric-specific evidence remain limited [70]. Most available studies are small, heterogeneous, and frequently lack adequate control groups, standardized protocols, or long-term follow-up, thereby limiting the strength and generalizability of conclusions for clinical practice.

Evidence suggests differential efficacy of FMT between UC and CD. In UC, pooled analyses summarized by Lahoud et al. demonstrate higher remission rates among patients treated with FMT compared with controls (37% vs. 18%), supporting a potential therapeutic role [71]. In contrast, findings in CD are inconsistent, with reported outcomes ranging from clinical and endoscopic remission to absence of measurable benefit. These discrepancies likely reflect differences in disease pathophysiology, anatomical involvement, microbial niches, and study design, underscoring the need for disease-specific evaluation rather than extrapolation across IBD subtypes.

Pediatric-specific data are particularly scarce. In a systematic review, Hsu et al. reported short-term clinical remission in 64.7% of pediatric CD patients one month following FMT, with a clinical response observed in 58.8% [72]. However, safety signals warrant careful consideration: serious adverse events were reported in 10% of cases, and overall adverse events occurred in 29%, though most were mild and self-limited [72]. In a pediatric population characterized by immune immaturity and frequent exposure to immunosuppressive therapies, these findings highlight the importance of cautious risk–benefit evaluation. Furthermore, the limited duration of follow-up in most pediatric studies precludes robust assessment of long-term efficacy, durability of remission, microbiome stability, and potential delayed complications associated with microbiota manipulation.

Broader evidence syntheses reinforce these uncertainties. The umbrella review by Malik et al., encompassing 16 systematic reviews and meta-analyses including pediatric patients, identified an association between FMT and improved remission and response rates in IBD—particularly in UC—compared with placebo or standard therapy [73]. However, these conclusions are largely driven by adult data, with pediatric outcomes often underreported or embedded within mixed-age cohorts. Consequently, extrapolation of adult findings to children remains methodologically and biologically uncertain.

Significant gaps persist in the evidence base. There is a notable lack of high-quality randomized controlled trials specifically designed for pediatric IBD, and very limited data evaluating long-term clinical outcomes, sustained microbiome reconstitution, and safety beyond the early post-transplant period [72,74]. Additionally, substantial heterogeneity exists across studies with respect to donor selection, stool preparation (fresh versus frozen), dosing frequency, route of administration, and outcome definitions, further limiting reproducibility and comparability.

Reflecting these limitations, current position statements from the NASPGHAN and the ESPGHAN do not recommend FMT as standard therapy for pediatric UC. Instead, its use is restricted to refractory cases and should be considered only in specialized centers, ideally within clinical trials or structured research protocols [17,50].

This cautious approach underscores the persistent gap between biological plausibility and high-quality clinical evidence. Well-designed, adequately powered pediatric trials with standardized methodologies and long-term follow-up are urgently needed to define the precise role of FMT in the management of pediatric IBD (Table 5).

It is important to emphasize that a substantial proportion of the available evidence on FMT in IBD is derived from adult populations, and caution is required when extrapolating these findings to pediatric patients. For example, the higher remission rates reported in pooled analyses (e.g., 37% vs. 18% in FMT-treated versus control groups) largely reflect data from adult cohorts and should be interpreted as illustrative of potential efficacy rather than as directly applicable to children. Pediatric-specific evidence remains limited to small, heterogeneous studies, often lacking randomized controlled designs and long-term follow-up. Consequently, reported remission and response rates in children are less robust and more variable, and differences in disease phenotype, immune maturation, microbiota composition, and treatment exposure may further limit direct comparability with adult populations. In this context, adult-derived data should be considered supportive of biological plausibility, while clinical recommendations in pediatric IBD must rely primarily on the limited but emerging child-specific evidence base.

Moreover, in contrast to UC, the evidence supporting FMT in CD—particularly in pediatric populations—remains extremely limited and inconclusive. Although CD accounts for approximately half of pediatric IBD cases and is characterized by a more complex, transmural, and segmental inflammatory process, few studies have specifically evaluated the role of microbiota-based therapies in this subgroup. Available data, largely derived from small adult cohorts, report highly variable outcomes ranging from clinical remission to no significant therapeutic benefit, reflecting heterogeneity in disease location, microbial niches, and host–microbiota interactions. Pediatric-specific evidence is even more scarce, consisting mainly of isolated case series and small observational studies, which preclude robust conclusions regarding efficacy. Furthermore, the distinct pathophysiological features of CD—including involvement of the small intestine, deeper tissue inflammation, and altered mucosal immune responses—may limit the effectiveness of luminal microbiota restoration strategies such as FMT. At present, there is insufficient evidence to support the routine use of FMT in pediatric CD, and its application should be considered experimental. Dedicated, well-designed studies focusing specifically on pediatric CD are urgently needed to determine whether selected patient subgroups may benefit from microbiota-targeted interventions.

### 3.5. Constipation and Irritable Bowel Syndrome

Chronic constipation and irritable bowel syndrome (IBS) are common functional gastrointestinal disorders in pediatric populations, characterized by significant symptom burden and impact on quality of life [75]. Increasing evidence implicates alterations in gut microbiota composition and function in their pathophysiology, including reduced microbial diversity, shifts in specific bacterial taxa, and altered production of metabolites such as short-chain fatty acids. These findings have provided a biological rationale for microbiota-directed therapeutic strategies, including FMT.

In constipation, dysbiosis has been associated with delayed intestinal transit, impaired neuromuscular function, and altered fermentation patterns. Preliminary studies suggest that FMT may improve stool frequency, consistency, and colonic transit time by restoring microbial balance and metabolic activity [76]. Proposed mechanisms include enhanced production of short-chain fatty acids, modulation of bile acid metabolism, and interaction with the enteric nervous system. However, available evidence remains limited, particularly in pediatric populations. Most data derive from small, uncontrolled studies or extrapolation from adult cohorts, with variable methodologies and inconsistent outcomes.

Similarly, IBS is increasingly recognized as a disorder involving complex interactions along the gut–brain–microbiota axis. Alterations in microbial composition may contribute to visceral hypersensitivity, low-grade mucosal inflammation, and dysregulated motility. In adult populations, randomized controlled trials have demonstrated mixed results regarding the efficacy of FMT in IBS, with some studies reporting symptom improvement [77,78], while others show no significant benefit despite microbiota changes [79]. More recent meta-analyses and randomized trials continue to report heterogeneous outcomes, likely reflecting differences in donor selection, administration routes, dosing regimens, and patient subtypes [80,81].

Pediatric data on FMT for IBS are extremely limited, and no robust randomized controlled trials have been conducted to date, although feasibility studies are ongoing [82]. Consequently, the role of FMT in this setting remains investigational. Current evidence does not support its routine use in children with functional gastrointestinal disorders, including constipation and IBS. Instead, FMT should be considered only within the context of well-designed clinical trials or highly selected refractory cases managed in specialized centers.

Overall, while FMT represents a biologically plausible intervention for constipation and IBS through restoration of microbial homeostasis and modulation of gut–brain signaling pathways, substantial gaps remain in the evidence base. Future research should focus on identifying microbiota signatures predictive of response, optimizing donor selection and treatment protocols, and evaluating long-term safety and efficacy in pediatric populations.

### 3.6. Allergic Colitis

Allergic colitis, most commonly presenting as food protein-induced allergic proctocolitis (FPIAP), is a non-IgE-mediated gastrointestinal disorder that primarily affects infants and young children [75]. It is characterized by colonic inflammation triggered by dietary antigens—most frequently cow’s milk protein—leading to symptoms such as rectal bleeding, mucus in stools, and irritability. Although the condition is generally benign and self-limiting, its pathogenesis is closely linked to immune dysregulation and altered intestinal barrier function.

Emerging evidence suggests that gut microbiota plays a pivotal role in the development of allergic colitis [83,84]. Infants with allergic colitis exhibit reduced microbial diversity, altered abundance of key commensal taxa (including Bifidobacterium and Lactobacillus species), and impaired production of immunomodulatory metabolites such as short-chain fatty acids. These alterations may contribute to an imbalance in T helper cell responses—particularly a skewing toward Th2-mediated immunity—thereby promoting allergic inflammation [85,86,87].

Given this mechanistic link, microbiota-targeted interventions have been proposed as potential therapeutic strategies. Probiotics and dietary modifications remain the cornerstone of management; however, interest in FMT has grown as a means of restoring microbial diversity and immune homeostasis. Preclinical studies and early clinical observations suggest that FMT may modulate immune responses by re-establishing microbial balance and promoting regulatory T-cell activity, thereby suppressing allergic inflammation [85,87]. Furthermore, microbiota restoration may enhance epithelial barrier integrity and reduce antigen translocation, which are key pathogenic mechanisms in allergic colitis.

Despite this strong biological rationale, clinical evidence supporting the use of FMT in allergic colitis remains limited, particularly in pediatric populations. Most available data derive from small case series, animal models, or extrapolation from studies in other allergic or inflammatory conditions. Recent reviews highlight that, although FMT has shown promise in modulating allergic diseases, robust clinical trials evaluating its efficacy and safety in allergic colitis are lacking [88]. Additionally, concerns regarding safety, donor selection, and long-term immune effects are particularly relevant in infants, in whom the microbiome is still undergoing critical developmental maturation.

At present, FMT cannot be recommended as standard therapy for allergic colitis and should be considered investigational. Its use should be restricted to research settings or highly selected refractory cases within specialized centers. Future studies are needed to clarify the role of microbiota modulation in allergic colitis, identify microbial signatures associated with disease and response to therapy, and determine the safety and long-term consequences of early-life microbiome manipulation.

### 3.7. Multi-Drug Resistant Organisms Decolonization

Intestinal colonization by MDRO, including vancomycin-resistant *Enterococcus* (VRE), carbapenem-resistant *Enterobacterales* (CRE), and other resistant Gram-negative bacteria, represents a major clinical concern in vulnerable pediatric populations, particularly in children with malignancies, transplant recipients, and those exposed to prolonged hospitalization or repeated broad-spectrum antibiotic therapy [89,90,91]. In these settings, the gut acts as a key reservoir for resistant pathogens and may facilitate subsequent invasive infections, especially in the presence of mucosal barrier disruption and immune suppression [92].

Because conventional antibiotic-based decolonization strategies are often ineffective and may further promote antimicrobial resistance, interest has grown in microbiota-based approaches aimed at restoring colonization resistance. FMT has therefore emerged as a biologically plausible strategy for MDRO decolonization, with the goal of re-establishing a diverse intestinal microbial ecosystem capable of suppressing resistant organisms through ecological competition, metabolite production, and restoration of mucosal homeostasis [93,94].

Available evidence suggests that FMT may reduce intestinal carriage of resistant organisms in selected patients, although reported efficacy remains heterogeneous. A prospective non-randomized study demonstrated that FMT could contribute to the clearance of intestinal colonization by CRE and VRE in high-risk patients [95]. In addition, even when complete microbiological eradication is not consistently achieved, FMT may provide clinically relevant benefits, including reduction in bloodstream infections, decreased antibiotic exposure, and improved clinical outcomes, suggesting that its effects may extend beyond pathogen elimination to broader microbiome restoration [96].

In pediatric populations, however, data remain extremely limited. A small case series reported successful MDRO decolonization following FMT in children undergoing HSCT, with associated microbiota reconstitution and favorable infectious outcomes [97]. More recent observational data in pediatric transplant recipients further support the potential role of FMT in this setting, although evidence remains limited to small cohorts and retrospective analyses [98].

Despite these promising findings, FMT for MDRO decolonization remains investigational. Existing studies are characterized by small sample sizes, heterogeneous patient populations, variability in FMT protocols, and inconsistent definitions of decolonization. Moreover, the durability of decolonization and long-term safety remain uncertain, particularly in immunocompromised children, in whom concerns persist regarding pathogen transmission, horizontal gene transfer, and unintended immunological effects [96].

At present, FMT cannot be recommended as standard therapy for MDRO decolonization in pediatric practice. Its use should be limited to highly selected cases and ideally performed within clinical trials or specialized centers with rigorous microbiological monitoring. Future research should focus on identifying optimal patient selection criteria, standardizing treatment protocols, and determining whether microbiota-based endpoints correlate with meaningful clinical outcomes, including reduction in invasive infections.

### 3.8. Neurodevelopmental Disorders

Neurodevelopmental disorders (NDDs), including autism spectrum disorder (ASD), attention-deficit/hyperactivity disorder (ADHD), and other cognitive and behavioral conditions, are characterized by impairments in social interaction, communication, and neurocognitive development [99,100]. Increasing evidence highlights the role of the gut–brain axis in their pathophysiology, emphasizing bidirectional interactions among the central nervous system, the enteric nervous system, immune pathways, and the intestinal microbiota [101,102].

Children with NDDs—particularly ASD—frequently present with gastrointestinal (GI) symptoms such as constipation, diarrhea, abdominal pain, and feeding disturbances, with reported prevalence rates exceeding 40–60% in some cohorts [103]. These symptoms are often associated with alterations in gut microbiota composition, including reduced microbial diversity, decreased abundance of beneficial taxa such as *Bifidobacterium* and *Prevotella*, and enrichment of pro-inflammatory or potentially pathogenic microorganisms [104]. Such microbial imbalances may influence neurodevelopment through several mechanisms, including modulation of systemic and mucosal immune responses, production of neuroactive metabolites (e.g., short-chain fatty acids, serotonin precursors), alteration of intestinal permeability (“leaky gut”), and activation of neuroinflammatory pathways [105].

Within this framework, FMT has emerged as a potential therapeutic strategy aimed at restoring microbial diversity and modulating gut–brain signaling [106]. One of the most widely cited approaches is microbiota transfer therapy (MTT), a modified FMT protocol involving antibiotic pre-treatment, bowel cleansing, and prolonged microbiota administration. In an open-label study including children with ASD, Kang et al. reported significant improvements in both GI and behavioral symptoms following MTT, with sustained benefits observed at two-year follow-up, including increased microbial diversity and normalization of key bacterial taxa [107,108]. These findings have generated considerable interest in the potential of microbiota-based interventions to influence neurobehavioral outcomes.

Subsequent studies have sought to validate these findings in more rigorous settings. A recent randomized, double-blind, placebo-controlled trial by Fecal Microbiota Transplantation Working Group investigators demonstrated that FMT was associated with improvements in GI symptoms in children with ASD, although effects on core behavioral outcomes were more variable and did not consistently reach statistical significance [109]. Similarly, other controlled and observational studies have reported heterogeneous results, with some demonstrating modest improvements in behavioral scales and others showing no significant benefit beyond placebo effects [110,111]. These discrepancies likely reflect differences in study design, including donor selection, route of administration (oral capsules versus colonoscopic delivery), dosing regimens, duration of treatment, and patient heterogeneity.

Mechanistically, FMT may exert beneficial effects by restoring microbial taxa involved in the production of short-chain fatty acids, particularly butyrate, which plays a critical role in maintaining intestinal barrier integrity and modulating neuroimmune signaling [112]. In addition, microbiota-derived metabolites may influence central nervous system function through modulation of the vagus nerve, endocrine pathways, and systemic cytokine profiles. Alterations in tryptophan metabolism and serotonin synthesis have also been implicated, providing a potential link between gut microbiota composition and behavioral regulation [112].

Despite these promising insights, several limitations constrain the current evidence base. Most pediatric studies are characterized by small sample sizes, short follow-up durations, and variability in outcome measures. Furthermore, the placebo response in neurodevelopmental disorders is known to be substantial, particularly in behavioral assessments, complicating interpretation of clinical efficacy. Importantly, long-term safety data are lacking, and the potential impact of microbiota manipulation on neurodevelopment, immune maturation, and metabolic programming during critical developmental windows remains incompletely understood [113].

Safety considerations are particularly relevant in children with NDDs, who may have underlying immune dysregulation or increased intestinal permeability. Although FMT is generally well tolerated, reported adverse events include transient gastrointestinal symptoms, and rare but serious complications such as infection transmission remain a concern, underscoring the importance of rigorous donor screening and standardized protocols [113].

At present, FMT cannot be recommended as a standard treatment for neurodevelopmental disorders in pediatric populations. Its use should be limited to clinical trials or highly selected cases, particularly when targeting severe, refractory gastrointestinal symptoms. Future research should prioritize large, multicenter randomized controlled trials with standardized methodologies, integration of microbiome and metabolomic profiling, and long-term follow-up to assess both efficacy and safety. Identification of microbiota signatures predictive of treatment response may further enable a precision medicine approach to microbiota-based interventions in neurodevelopmental disorders.

Overall, while FMT represents a biologically plausible and potentially transformative approach for modulating the gut–brain axis, current evidence remains preliminary, and its role in pediatric neurodevelopmental disorders requires further rigorous investigation.

## 4. Clinical Implications, Safety Considerations, and Future Directions of Fecal Microbiota Transplantation in Pediatric Practice

The intestinal microbiota represents a central regulator of host defense in neonates and children, exerting critical effects on immune maturation, colonization resistance, metabolic homeostasis, and preservation of epithelial barrier integrity [4,5,6,7,8]. Disruption of this complex and dynamic ecosystem—most commonly resulting from exposure to broad-spectrum antibiotics, cytotoxic chemotherapy, or prolonged hospitalization—has been consistently associated with adverse infectious outcomes, including bloodstream infections and increased treatment-related morbidity [7,8,13]. These associations are particularly pronounced in vulnerable pediatric populations, such as preterm infants and children undergoing oncologic therapies, in whom microbiota perturbation may amplify immune dysregulation and susceptibility to opportunistic pathogens [55,56,57].

Growing evidence suggests that microbiota-directed strategies—including optimized antimicrobial stewardship, targeted probiotic administration, and FMT—may promote restoration of microbial diversity and functional resilience, thereby mitigating infection risk and improving clinical outcomes [21,22,45]. In this context, antimicrobial stewardship represents a cornerstone intervention, as inappropriate or prolonged antibiotic exposure is the primary driver of dysbiosis, CDI, and microbiota disruption in both general pediatric and immunocompromised populations. Rational antibiotic use not only reduces the incidence of CDI but also preserves microbiome integrity, potentially decreasing the need for downstream interventions such as FMT.

Despite increasing clinical enthusiasm, the current evidence base for FMT in pediatrics remains constrained by significant methodological and translational limitations. High-quality, multicenter randomized controlled trials are scarce, and much of the available literature derives from observational studies or single-center cohorts, frequently limited to narrowly defined indications such as CDI [30,50]. Substantial heterogeneity in intervention design—including variability in microbial composition, strain selection, dosing regimens, timing relative to antibiotic exposure, preparation methods, and duration of therapy—further complicates interpretation and limits standardization [18,20,23].

Safety considerations represent a critical aspect of FMT implementation in children and require a more structured evaluation. Reported adverse events are generally mild and self-limited, including abdominal discomfort, transient diarrhea, bloating, and low-grade fever. However, more serious complications—although rare—have been described, including bacteremia, transmission of multidrug-resistant organisms, and sepsis, particularly in immunocompromised patients [18,24]. The frequency and severity of adverse events appear to vary according to patient population, route of administration, and underlying clinical condition. Notably, children with malignancies, transplant recipients, and those with severe immunosuppression may be at increased risk of complications. Table 6 summarizes adverse events associated with FMT across pediatric indications.

The route of FMT administration represents an additional key consideration in pediatric practice. While colonoscopic and nasoenteric delivery have historically been the most commonly used approaches, oral capsule formulations are emerging as a particularly attractive alternative in children, offering a non-invasive, well-tolerated option that avoids procedural risks such as sedation, endoscopy-related complications, and patient discomfort. Preliminary studies in both adult and limited pediatric populations suggest that capsule-based FMT may achieve comparable efficacy to traditional delivery routes, with high acceptability and improved feasibility in outpatient settings [17,21,52]. However, pediatric-specific data remain limited, and further studies are needed to define optimal dosing, formulation, and age-appropriate protocols.

Long-term safety data remain particularly limited, especially in immunocompromised children. In this population, microbiota-based interventions raise unresolved concerns regarding pathogen transmission, horizontal gene transfer, and unintended immunologic consequences [18,24]. Although FMT has demonstrated efficacy in rCDI, current evidence remains insufficient to support its routine use in pediatric patients with malignancies or severe immunosuppression [50,66]. Existing data are largely derived from small retrospective series or extrapolated from adult cohorts, with minimal representation of pediatric oncology patients and an almost complete absence of neutropenic children [59,65].

Additional uncertainty arises from heterogeneity in patient selection criteria, definitions of immunocompromise, FMT preparation (fresh versus frozen), routes of administration, and outcome measures [18,23]. Such variability hampers meaningful comparison across studies and contributes to the cautious stance reflected in current clinical practice guidelines [50,66]. Furthermore, most studies lack extended follow-up, limiting the ability to assess delayed adverse events, including donor-derived infections or long-term alterations in immune homeostasis—issues of particular concern in children receiving cytotoxic or immunomodulatory therapies [24].

The systematic exclusion of pediatric oncology patients from randomized trials further widens this evidence gap. While exclusion criteria are often justified by theoretical safety concerns—such as increased intestinal permeability and bacterial translocation—they also perpetuate reliance on indirect or adult-derived evidence [59]. At the same time, emerging data highlight the profound impact of microbiota composition on cancer treatment outcomes, including chemotherapy toxicity, immune reconstitution, and response to advanced therapies such as CAR-T cells and HSCT, reinforcing the biological rationale for microbiota-targeted interventions in this population.

Looking forward, integration of microbiome profiling into clinical practice represents a major opportunity to advance precision medicine approaches in this field. Metagenomic sequencing, metabolomic profiling, and 16S rRNA-based analyses may enable identification of patients at high risk of dysbiosis, prediction of response to FMT, and monitoring of microbial engraftment following transplantation. In addition, early detection of microbiota alterations could allow preemptive interventions before clinical manifestations such as CDI recurrence or IBD flare occur.

Future research should follow a prioritized and structured roadmap. In the near term, achievable goals include standardization of FMT protocols, harmonization of outcome definitions, and incorporation of safety reporting frameworks within ongoing pediatric trials. Priority populations for prospective studies should include high-risk groups such as children with rCDI, pediatric oncology patients, and HSCT recipients, in whom the burden of dysbiosis-related complications is greatest. In parallel, longer-term research should focus on mechanistic studies integrating multi-omics approaches to identify microbial and metabolic signatures predictive of response, as well as the development of targeted, next-generation microbiota-based therapies. Recent evidence highlighting the interplay between gut microbiota and cancer treatment outcomes in pediatric leukemia provides a strong rationale for prioritizing these populations in future FMT trials and microbiome-based intervention studies.

Ultimately, a deeper understanding of host–microbiota–pathogen interactions across developmental stages will be critical to advancing precision microbiome-based strategies aimed at improving infectious outcomes, minimizing treatment-related toxicity, and reducing the growing burden of antimicrobial resistance in pediatric care [4].

**Figure 1:**
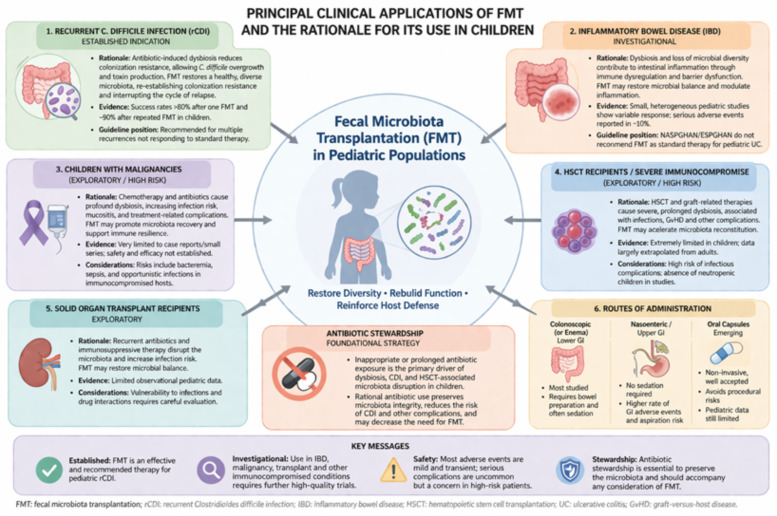
Fecal microbiota transplantation (FMT) in pediatric population. FMT, fecal microbiota transplantation; rCDI, recurrent *Clostridioides difficile* infection; CDI, *Clostridioides difficile* infection; IBD, inflammatory bowel disease; UC, ulcerative colitis; HSCT, hematopoietic stem cell transplantation; GvHD, graft-versus-host disease; GI, gastrointestinal.

Figure 1 summarizes the main clinical indications, safety considerations, and research priorities for FMT in pediatric practice, highlighting its established role in recurrent CDI and its investigational status in other dysbiosis-associated conditions.

## 5. Conclusions

FMT should currently be considered an established therapeutic option in children with rCDI who meet guideline-based criteria, particularly after failure of appropriate antibiotic regimens. In contrast, its use for immunocompromised population, IBD, allergic colitis, functional gastrointestinal disorders, MDRO decolonization, and neurodevelopmental disorders remains investigational and should be limited to specialized centers or clinical trials.

Clinicians should carefully evaluate patient selection, donor screening, route of administration, and short- and long-term safety, especially in infants, oncology patients, transplant recipients, and other immunocompromised children. Standardized protocols, harmonized clinical endpoints, and long-term follow-up are required before FMT can be routinely recommended beyond rCDI. Until stronger pediatric evidence is available, antimicrobial stewardship and microbiota-preserving strategies should remain central to preventing dysbiosis and reducing the need for microbiota-restorative interventions.

## Figures and Tables

**Table 1:** Epidemiology and major risk factors for pediatric *Clostridiodes difficile* infection.

Aspect	Key Findings	Reference
Incidence trend	Increasing over the last two decades in both hospital- and community-associated settings	[28,32,33,34]
High-risk pediatric populations	Oncology patients, transplant recipients, children with IBD, immunocompromised patients	[34,35,36]
Recurrence risk	Higher in children with chronic disease and immunosuppression	[34,35,36]
Healthcare vs. community	Predominantly healthcare-associated, but rising community-associated cases	[28,32,33,34]
Age-related features	High asymptomatic colonization in infancy; increased symptomatic disease in older children	[37]

**Table 2:** Age-dependent patterns of colonization and disease expression.

Age Group	Colonization Rate	Clinical Manifestations	Pathophysiological Features
Neonates (0–1 week)	~0% initially; increases by end of first week	Asymptomatic	Immature microbiota; limited toxin receptor expression
Infants (<12 months)	Up to 70%	Rarely symptomatic	Resistance to toxins A/B; protective microbiota
Toddlers (12–24 months)	Declining	Transition phase	Microbiota maturation
Older children	Low	Symptomatic *Clostridiodes difficile* infection, colitis	Adult-like microbiota; susceptibility to toxin injury

**Table 3:** Key microbiota alterations associated with pediatric *Clostridioides difficile* infection.

Microbial Feature	Change During CDI	Clinical Implication	References
Species richness	Decreased	Loss of colonization resistance	[43,44]
*Firmicutes*	Depleted	Reduced butyrate production	[44]
*Bacteroidetes*	Depleted	Impaired metabolic homeostasis	[7,8,44]
*Actinobacteria*	Reduced	Loss of protective taxa (e.g., *Bifidobacterium*)	[44]
*Proteobacteria*	Expanded	Pro-inflammatory milieu	[44]
Short-chain fatty acids	Reduced (especially butyrate)	Barrier dysfunction, immune dysregulation	[7,8,44]

**Table 4:** Theoretical rationale and limitations of fecal microbiota transplantation in pediatric oncology patients.

Aspect	Description
Reasons for exclusion from trials	Risk of bacterial translocation and donor-derived infections
Differences compared to adults	Different chemotherapy regimens, antimicrobial exposure, and comorbidities
Theoretical benefits	Restoration of *Bacteroidetes* and *Firmicutes;* possible reduction in multidrug-resistant organisms
Current status of fecal microbiota transplantation	Experimental option after immunosuppression

**Table 5:** Evidence on fecal microbiota transplantation in pediatric inflammatory bowel disease.

Domain	Key Findings	Notes/Limitations	References
IBD epidemiology	~25% of IBD diagnoses occur before age 20; incidence rising in pediatric populations	Chronic disease with long-term morbidity and extra-intestinal manifestations	[67,68,69,70]
Rationale for FMT	Targets intestinal dysbiosis by restoring microbial diversity and functional balance	Strong biological plausibility, but clinical translation remains uncertain	[12,17,50,72,74]
Overall evidence quality	Limited and heterogeneous, especially in pediatric cohorts	Small sample sizes, lack of controls, non-standardized protocols, short follow-up	[50,72,73,74]
FMT efficacy in UC	Higher remission rates with FMT vs. controls (37% vs. 18%)	Data largely driven by adult studies	[71,73,74]
FMT efficacy in CD	Inconsistent and weak evidence; outcomes range from remission to no benefit	Likely reflects disease-specific pathophysiology and microbial differences	[71,73,74]
Pediatric-specific outcomes	Short-term remission: 64.7%; clinical response: 58.8% at 1 month	Follow-up duration generally insufficient to assess durability	[72]
Safety in pediatric patients	Serious adverse events: 10%; overall adverse events: 29% (mostly mild, self-limited)	Safety concerns amplified by immune immaturity and immunosuppression	[2,72]
Umbrella review findings	Association with improved remission and response, especially in UC	Pediatric data underreported or embedded in mixed-age cohorts	[73]
Methodological variability	Donor selection, stool preparation, dosing, and administration routes vary widely	Limits reproducibility and cross-study comparisons	[17,18,23,50,72,73,74]
Guideline recommendations	FMT not recommended as standard therapy for pediatric UC	Restricted to refractory cases in specialized centers or clinical trials	[17,50,74]

CD, Crohn’s disease; IBD, inflammatory bowel disease; FMT, fecal microbiota transplantation; UC, ulcerative colitis.

**Table 6:** Adverse events associated with fecal microbiota transplantation across pediatric indications.

Population/Indication	Adverse Events	Severity	Frequency	Notes	References
Recurrent CDI	Abdominal pain, diarrhea, bloating, fever	Mostly mild	Common	Best-studied indication	[17,23,24,50,52,53]
IBD (UC/CD)	GI symptoms, disease flare, fever	Mild–moderate; some severe	29% overall AE; 10% serious	Higher risk due to inflammation	[24,72,73,74]
Oncology patients	GI symptoms; risk of bacteremia/sepsis	Potentially severe	Rare but unclear	Immunosuppression	[59,65,66]
HSCT recipients	Fever, infection risk	Moderate–severe potential	Limited data	High-risk population	[60,61,65,66]
Solid organ transplant	GI symptoms	Mostly mild	Unknown	Limited pediatric data	[62,63,64,65]
MDRO decolonization	GI symptoms, infection risk	Mild–moderate	Variable	Heterogeneous evidence	[89,90,91,92,93,94,95,96,97,98]
Neurodevelopmental disorders	GI symptoms	Mild	Common	Generally well tolerated	[104,107,108,109,110,111,112,113]
Allergic colitis	GI symptoms	Mild	Limited data	Mostly theoretical	[83,84,85,86,87,88]
Constipation/IBS	GI discomfort	Mild	Variable	Limited pediatric data	[75,76,77,78,79,80,81,82]

## Data Availability

No new data were created or analyzed in this study.
